# Cardiovascular risk factors in children and adolescents with subclinical hypothyroidism: A protocol for meta-analysis and systematic review

**DOI:** 10.1097/MD.0000000000020462

**Published:** 2020-07-31

**Authors:** Huan DENG, Shan ZHOU, Xian WANG, Xianliang QIU, Qing WEN, Shiyu LIU, Qiu CHEN

**Affiliations:** a School of Clinical Medicine, Hospital of Chengdu University of Traditional Chinese Medicine; b Department of Endocrinology, Hospital of Chengdu University of Traditional Chinese Medicine, Chengdu, P.R. China.

**Keywords:** adolescents, cardiovascular, children, lipid, subclinical hypothyroidism

## Abstract

**Introduction:**

Subclinical hypothyroidism (SH) is serum thyrotropin (TSH) slightly increased, while serum free thyroxine (FT4) levels are normal, and patients may have no abnormal symptoms, or only slight hypothyroidism, there are many studies proving that SH does increase cardiovascular risk in adults. However, there are few studies in children and adolescents. In order to explore whether children with subclinical hypothyroidism have a higher cardiovascular risk, we designed this meta-analysis.

**Methods:**

The protocol of this systematic review and meta-analysis was registered on the NPLASY (No. 202040182). The following electronic databases will be searched from the inception through the present to find studies that live up to our standard: PubMed, EMBASE, Web of Science, Cochrane Library, CNKI, Wanfang, and VIP. And we will include case-control studies, cohort studies, and cross-sectional studies. For including study, we will use the Newcastle-Ottawa Scale to evaluate their quality. Then 2 researchers will independently extract the required information. *I*^2^ statistics and subgroups will be used to analyze heterogeneity. We conduct a sensitivity analysis by excluding literature successively. When the system review contains >10 articles, Egger test will be conducted to evaluate publication bias.

**Results:**

From this study, we will assess the cardiovascular risk of children and adolescents with SH from multiple perspectives.

**Conclusion:**

The conclusion of this paper will provide evidence for cardiovascular risk of SH children and provide basis for prevention and treatment of SH.

**Ethics and dissemination:**

This meta-analysis does not collect personal primary data, so there is no need for formal moral recognition. The results of the system review will be presented to national and international conferences for publication.

## 1 Introduction

Subclinical hypothyroidism (SH) is serum thyrotropin (TSH) slightly increased, while serum free thyroxine (FT4) levels are normal, and patients may have no abnormal symptoms, or only slight hypothyroidism.^[1]^ When the serum TSH level is between 4.5 and 10 mIU/L, the degree of subclinical hypothyroidism is mild, and when it is >10 mIU/L, the disease is defined as severe.^[1]^

According to the investigation, the incidence of SH in adults is about 3% to 15%,^[2]^ what is more, it has a high chance of developing overt hypothyroidism. However, due to the lack of research data, the prevalence of children seems to be relatively lower, <2%.^[3]^

As we all know, thyroid hormone has anti-atherosclerotic effects. A large and growing body of research has confirmed that hypothyroidism accelerates the atherosclerotic process in several ways, such as lipid profile, blood pressure, and endothelial function, it also has a direct impact on cardiovascular function.^[4]^ At the same time, there are many studies proving that SH does increase cardiovascular risk in adults. It includes the following aspects: left ventricular diastolic function,^[5]^ arterial function,^[6–8]^ lipid profile,^[9]^ and more. However, there are few studies in children and adolescents. According to some studies, SH in children and adolescents is benign, palliative, also, it does not easily develop into significant hypothyroidism,^[10]^ but whether it will affect children's health remains a mystery. So, it's necessary to research it, because if there's a cardiovascular risk factor in childhood, there's a greater cardiovascular risk in adulthood.^[11]^

As far as we know, there is no systematic review about cardiovascular risk factors in children and adolescents with SH. In order to explore whether children with subclinical hypothyroidism have a higher cardiovascular risk, we designed this meta-analysis.

## 2 Methods

Preferred Reporting Items for Systematic Reviews and Meta-Analyses (PRISMA)^[12]^ Statement will run through our whole study, and this protocol will follow the PRISMA protocols (PRISMA-P) 2015 statement.^[13]^ The protocol of this systematic review and meta-analysis was submitted on the International Platform of Registered Systematic Review and Meta-analysis Protocols (INPLASY) (No. 202040182) which could be available on https://inplasy.com/.

### 2.1 Eligibility criteria

#### 2.1.1 Participants

The study includes children and adolescent participants under the age of 18. Patients who receive L-thyroxine (L-T4) replacement therapy will be excluded.

#### 2.1.2 Control and exposed groups

To control the potential bias and have a comparison, only those with the control group can be included in this review. The exposed group will be children and adolescents with SH, compared with healthy participants with normal thyroid function.

#### 2.1.3 Outcomes

Lipid profile will be the primary outcome, including serum levels of total cholesterol (TC), high-density lipoprotein cholesterol (HDL-C), triglycerides (TG), and low-density lipoprotein cholesterol (LDL-C). The secondary outcomes (if documented) will include other cardiovascular risk factors, like intima-media thickness (IMT), insulin resistance, and tissue Doppler imaging.

#### 2.1.4 Study design

Our review will contain the study described at least 1 cardiovascular risk factor associated with subclinical hypothyroidism in children. We will include case-control studies, cohort studies, and cross-sectional studies. Conference abstracts, critical papers, system reviews, organizational reports, research papers will be excluded.

### 2.2 Information sources and search strategy

The following electronic databases will be searched from the inception through the present to find studies that live up to our standard: PubMed, EMBASE, Web of Science, Cochrane Library, CNKI, Wanfang, and VIP. And we will screen reference lists of identifying studies. Two experienced researchers (HD and QW) worked out the research strategy independently. Our review is limited to English articles or articles that can be translated into English. We will use the following keywords to search for literature: children, adolescents, subclinical hypothyroidism, cardiovascular, lipids, insulin resistance, and intima-media thickness. Taking the strategy of retrieving PubMed database as an example, the search strategy is as follows:

#1 children OR child OR Adolescents OR Adolescent OR Adolescence OR Teens OR Teen OR Teenagers OR Teenager OR Youth OR Youths OR Adolescents, Female OR Adolescent, Female OR Female Adolescent OR Female Adolescents OR Adolescents, Male OR Adolescent, Male OR Male Adolescent OR Male Adolescents OR pediatric OR pediatrics
#2 subclinic OR subclinical OR subclinically OR subclinicals
#3 hypothyroidal OR hypothyroidic OR hypothyroidism OR hypothyroidism OR hypothyroid OR hypothyroidisms OR hypothyroids
#4 cardiovascular OR lipids OR lipid OR cholesterol OR Epicholesterol OR triglyceride OR triacylglycerol OR triacylglycerols OR Insulin Resistance OR Resistance, Insulin OR Insulin Sensitivity OR Sensitivity, Insulin OR intima-media thickness OR Carotid Intima Media Thickness OR Intima-Media Thickness, Carotid OR Atherosclerosis OR Atheroscleroses OR Atherogenesis
#5 #1 AND #2 AND #3 AND #4

### 2.3 Study selection

First, the first batch of documents that meet the standards are determined through the selection of titles and abstracts, then further screening is 2 reviewers selecting by reading the full text and recording the cause of excluded literature. If 1 standard research is not available online, we will send an email to the author to get the full text or the required data. Finally, we will import all research articles that are compliant with inclusion criteria into Endnote X9 and remove duplicate research. Also, in this meta-analysis, the same research published in multiple publications will be regarded as one. We will consensus with a third researcher (XLQ) to resolve the differences.

### 2.4 Study quality

The Newcastle-Ottawa Scale (NOS)^[14]^ will be used to assess the bias of the studies included in this meta-analysis. This scale is evaluated from 3 aspects, including object selection, comparability, outcome, and exposure. By comparing this scale, all studies will be divided into 3 quality grades based on score: high, medium, and low, and high quality articles score 7 to 9 stars, medium quality is 4 to 6 stars, and the lowest is 0 to 3 stars.

### 2.5 Data extraction and synthesis

For qualified articles, the 2 researchers will independently extract the required information and record the data in the standard extraction table, a third researcher will help them when there is a dispute between them. The table contains the following data: author, study type, study design, study time, study area, number of participants, baseline characteristics (sex, age, body mass index [BMI], TSH, FT4, etc), and clinical outcomes (TC, HDL-C, LDL-C, TG, IMT, insulin resistance, etc). The overall quality of extracted data will be assessed by using the Grading of Recommendations, Assessment, Development and Evaluation (GRADE)^[15]^ assessment tool.

RevMan, version 5.3 software will be used to analyze the data. We assessed heterogeneity using *I*^2^ statistics. If *I*^2^ statistic exceeded 50%, the random effect model will be used to summarize the study, and if it is not >50%, the fixed effects model will be applied. In this study, standardized mean deviation (SMD) and 95% confidence interval will be used for continuous results, and relative risk (RR) and 95% confidence interval will be used for dichotomy results. In addition, when *I*^2^ ≥ 50%, subgroup analysis will be used to explore potential heterogeneity according to participants and exposure characteristics mentioned above. We conduct the sensitivity analysis by excluding literature successively. When the system review contains >10 articles, the Egger test^[16]^ will be conducted to evaluate publication bias.

## 3 Discussion

We designed this meta-analysis because the jury is still out on the cardiovascular risk of children with subclinical hypothyroidism. This study allows us to understand the relationship between children and adolescents with subclinical hypothyroidism and some cardiovascular risk factors, it is helpful to enhance our understanding of SH in children and provide a reference for the treatment of them.

## Author contributions

**Idea conception:** Huan DENG, Shan ZHOU.

**Literature retrieval:** Huan Deng, Qing Wen, Xianliang QIU.

**Methodology:** Huan DENG, Xian WANG.

**Review guarantor:** Qiu CHEN.

**Writing – initial manuscript:** Huan DENG, Xian WANG.

All authors participated in the revision of the manuscript and agreed to publish it.
